# Quercetin associated with dimethylsulfoxide has a curative effect on experimental colon anastomosis injury^1^

**DOI:** 10.1590/s0102-865020200060000002

**Published:** 2020-07-08

**Authors:** Ufuk Demir, Mustafa Edremitlioğlu, Emel Kandaş, Müşerref Hilal Şehitoğlu, Nihal Kılınç

**Affiliations:** I MSc, Department of Physiology, Canakkale Onsekiz Mart University, Canakkale, Turkey. Acquisition, analysis and interpretation of data; statistics analysis, technical procedures, manuscript preparation.; II MD, Department of Physiology, Canakkale Onsekiz Mart University, Canakkale, Turkey. Conception and design of the study, manuscript preparation and writing, critical revision, final approval.; III MSc, Department of Physiology, Canakkale Onsekiz Mart University, Canakkale, Turkey. Acquisition of data, technical procedures.; IV PhD, Department of Biochemistry, Canakkale Onsekiz Mart University, Canakkale, Turkey. Analysis and interpretation of data, technical procedures.; V MD, Department of Pathology, Canakkale Onsekiz Mart University, Canakkale, Turkey. Analysis and interpretation of data, histopathological examinations.

**Keywords:** Quercetin, Anastomosis, Surgical, Antioxidants, Rats

## Abstract

**Purpose:**

To examine the effects of quercetin on healing of experimental colon anastomosis injury in early and late period.

**Methods:**

Eighty male Wistar-Albino rats were divided into 8 groups. For all groups, left colons of the rats were resected and for the rest end-to-end anastomosis was performed. Two of the groups for which the experiment protocol was ended on the 3rd and 7th day following the anastomosis were not administered with either quercetin or dimethylsulfoxide DMSO, whereas two other groups were administered with DMSO only, and four other groups were administered with quercetin dissolved in DMSO in doses of 20 and 100 mg/kg during the protocol. At the end of the study, anastomosis line was resected, histopathological evaluation was performed and bursting pressure, malondialdehyde, superoxide dismutase, catalase, and hydroxyproline levels were measured.

**Results:**

Quercetin significantly increased hydroxyproline, superoxide dismutase, catalase levels, histopathological healing score, bursting pressure values and decreased malondialdehyde level in early period. It also significantly increased superoxide dismutase, catalase, and hydroxyproline levels and decreased malondialdehyde level in late period.

**Conclusion:**

It was seen that quercetin speeds up the injury healing process and reveals an antioxidant effect, specifically in early period.

## Introduction

Colon cancer is ranked third among all types of cancers as regards to incidence, whereas it is the second leading cause of cancer-related deaths^1^. Surgical intervention has an important role in treatment methods. Colorectal surgery is being widely used in colorectal cancers as well as ischemic colitis, ulcerative colitis, Crohn disease, mechanical intestinal obstruction, trauma, and recurrent diverticulitis^2^. Today, despite the developments in surgical techniques and the application of new technologies such as robotic surgery, there are still cases which end up with death after colon anastomosis when the anastomosis area cannot heal completely and there is a leakage^3,4^. Ischemia, anemia, malnutrition, tension in anastomotic line, surgical technique, localized infection, and obstruction in distal anastomosis may be listed among the possible causes of anastomotic leaks^3,5^. Whatever the cause, anastomotic leaks can still be observed at a rate of 1-19.2% after colorectal surgery interventions despite the developments in surgical techniques^3,4,6^.

Full and rapid healing of the anastomosis injury is very important in reducing the risk of leakage after surgery. Although several methods and materials have been used to speed up the healing of the injury and to increase the strength of the tissue newly developing, a method fully accepted could not be defined yet.

One of the molecules effective in healing of injuries is quercetin^7-9^. Quercetin is a polyphenolic compound that has the structure of 3,5,7,3’,4’-penta hydroxy flavonol^10^. Quercetin has a strong antioxidant effect. It has also effects on cleaning free radicals such as superoxide, hydroxyl, peroxyl, alcoxyl, and lipid peroxyl, inhibition of cyclooxygenase and lipoxygenase enzymes and metal chelation^11-13^. In literature studies, it was shown that quercetin has a beneficial effect on healing of skin injuries^5^. This effect of quercetin is thought to be coming from its antioxidant feature. As a matter of fact, there are studies that support the importance of antioxidant effect on healing of injuries^14-16^. Furthermore, it was also shown that antioxidant effect is very important in healing of injuries after colon surgeries^17-20^. When the beneficial effects of antioxidants on healing of injuries and the antioxidant effect of quercetin come together, it suggests that quercetin can contribute to the healing of colon anastomotic injury and can prevent the complications that occur after leakage. Therefore, we studied how quercetin affects the healing of injury after colon anastomosis by using two different doses of quercetin. For this purpose, we administered quercetin intraperitoneally to rats, which were applied colon anastomosis, beginning from the date of anastomosis. We examined the effects of the treatment on healing of surgical injury and antioxidant enzyme activities on early period (third day after anastomosis) and late period (seventh day after anastomosis).

## Methods

### *Experimental animals and environment*

This study was realized at Çanakkale Onsekiz Mart University Experimental Researches Implementation and Research Center, Medical Faculty Physiology Laboratory, and Medical Faculty Pathology Laboratory. The project was found in compliance with ethical board directives and approved by Çanakkale Onsekiz Mart University Ethical Board of Animal Testing (Approval No. Of the Ethical Board: 2013/09-09, 2015/08-26).

In this study, 80 male Wistar-Albino rats (ÇOMÜDAM, Çanakkale, Turkey) of 230-260 gr were used. During the experiment period, the rats were observed in the laboratory with 20±2 ºC constant room temperature, 50% humidity, and 12 hours day and night cycles. For their nutrition standard animal feed and tap water were used.

### *Experimental groups*

The rats were divided into 8 groups, each group having 10 rats.

(1) Control 3 days (C3 group)

(2) Control 7 days (C7 group)

(3) Dimethylsulfoxide (DMSO) control 3 days (DMSO3 group)

(4) DMSO control 7 days (DMSO7 group)

(5) Quercetin 20 mg/kg 3 days (Q3/20 group)

(6) Quercetin 100 mg/kg 3 days (Q3/100 group)

(7) Quercetin 20 mg/kg 7 days (Q7/20 group)

(8) Quercetin 100 mg/kg 7 days (Q7/100 group)

Colon anastomosis was performed in all groups. The healing of injury was observed on the third day of surgery for 3 days groups, and on the seventh day of surgery for 7 days groups. After surgery, control groups were applied with physiological saline solution, DMSO groups were applied with DMSO, and quercetin groups were applied with quercetin (Sigma-Aldrich Co., St. Louis, USA) dissolved in DMSO (20 and 100 mg/kg) (Sigma-Aldrich Co., St. Louis, USA) with injections intraperitoneally (IP)^21,22^.

### *Surgical procedure*

Animals used in this study were anesthetized by intramuscular injection of a mixture of Xylazine (5 mg/kg Rompun®, Bayer Healthcare, Kansas, USA) and Ketamine (50 mg/kg-Ketalar®, Pfızer-Zentiva, Lüleburgaz, Turkey) following a 12 hour fasting. All stages of the operation were performed under aseptic circumstances.

Abdomens of rats were opened by a midline cut. Cecum and colon were taken out. A 1 cm piece of the left colon was cut and taken out from the colorectal junction. Then, colon anastomosis was made end-to-end with a 6/0 polypropylene suture. After the operation, fascia and skins of the rats were closed continuously with 3/0 silk suturing. To prevent dehydration, 5 ml ringer lactate solution was applied subcutaneously^19^. The rats were allowed to be fed freely after the effects of the anesthesia wore off.

### *Actions carried out after the anastomosis*

Subjects were anesthetized by using ether on the third and the seventh day. The animals were sacrificed after blood samples were taken by cardiac puncture. Abdomens of the subjects were opened and 2 cm distal and 2 cm proximal of the anastomosis and intestine including the anastomosis line were resected and anastomosis bursting pressure at the anastomosis line was measured. After this measurement, colon including 0.5 cm distal and 0.5 cm proximal of the anastomosis was resected. After that, half of the anastomosis line of the rats was put into a – 80°C freezer for measurement of malondialdehyde (MDA) level, superoxide dismutase (SOD) activity, catalase (CAT) activity, and hydroxyproline level; other half was kept in a formol solution for pathological examination.

### *Anastomosis bursting pressure measurement*

Two cm distal and 2 cm proximal of the anastomosis and intestine including the anastomosis line were resected. After the available fecal content was washed with physiological saline solution and removed, one end of the colon was attached to the infusion pump and the other end was attached to the pressure transducer (Biopac MP 35 Data Acquisition System, Ankara, Turkey) by 2/0 silk suturing. After the distal catheter was attached to the pressure transducer in the data collecting system, physiological saline solution infusion was made from the proximal catheter at a 4 ml/min speed via infusion pump. Meanwhile, pressure level reached during the bursting of anastomosis location was determined by recording changes in pressure in the intestinal segment continuously.

### *Measurement of *hydroxyproline* level*

For hydroxyproline measurement, tissues were weighed and taken into eppendorf tubes and 100 µl distilled water was added. After 100 µl and 12M concentrated hydrochloric acid was added, they were kept in a laboratory oven (Thermo Scientific Heratherm oven OGS60, Langenselbold, Germany) at 120^ο^C for 3 hours. Cooled samples were centrifuged at 4000 rpm for 10 min. The supernatants obtained were transferred to eppendorf tubes readily prepared for activity measurement and hydroxyproline was measured spectrophotometrically at 560 nm as defined in the kit prospectus (Sigma-Aldrich® Hydroxyproline Assay Kit MAK008, St. Louis, USA). The results were determined as μg/mg ww (wet weight).

### *Measurement of malondialdehyde (MDA) level*

Tissue MDA levels were determined according to the spectrophotometric method defined by Ohkawa *et al*.^23^. Samples taken were weighed by precision scales and then their homogenates were prepared. Tissues were weighed in 100 mg for the homogenization process. 50 mM (pH=7) phosphate buffer was added to make a dilution at a rate of 1/10 (m/v). In order to prevent contamination, 1 mg butyl hydroxytoluene (BHT) was added per 10 mg sample. Then it was homogenized in a ball homogenizer (Retsch MM400, Retsch GmbH, Haan, Germany) at 30 Hz frequency for 10 min. Samples taken from the homogenizer were centrifuged at 4000 rpm for 10 min. The supernatants obtained were transferred to eppendorf tubes readily prepared for activity measurement. Tissue MDA determination was made by spectrophotometric measurement at 532 nm of a pink complex generated by MDA, which is the last product of lipid peroxidation that occurs as a result of incubation of tissue homogenate in hot water bath for one hour, with thiobarbituric acid (TBA) (Sigma-Aldrich Co., St. Louis, USA).

### *Measurement of superoxide dismutase (SOD) activity*

Samples taken were weighed by precision scales in accordance with SOD kit (Cayman Chemical Company Superoxide Dismutase Assay Kit 706002, E. Ellsworth Rd-Ann Arbor, MI, USA) procedure and their homogenates were prepared. Tris-HCI buffer (pH=7.4) was added to tissue samples to make a dilution at a rate of 1/10 (m/v). The samples were homogenized in a ball homogenizer (Retsch MM400) at 30 Hz frequency for 10 min by being kept in their coldness. The tubes taken from the homogenizer were centrifuged at 4000 rpm for 10 min. SOD activity was measured spectrophotometrically at 440-460 nm on the supernatants obtained as defined in the kit prospectus.

### *Measurement of catalase activity*

Samples taken were weighed by precision scales in accordance with catalase kit (Cayman Chemical Company Catalase, E. Ellsworth Rd-Ann Arbor, MI, USA) procedure and their homogenates were prepared. 50 mM (pH=7) phosphate buffer was added to tissue samples to make a dilution at a rate of 1/10 (m/v). The samples were homogenized in a ball homogenizer (Retsch MM400) at 30 Hz frequency for 10 min. The tubes taken from the homogenizer were centrifuged at 4000 rpm for 10 min. Catalase activity was measured spectrophotometrically at 540 nm on the supernatants obtained as defined in the kit prospectus. The results were determined as U/mg ww.

### *Histopathological examination*

Histopathological evaluation was performed by a pathologist who did not have the knowledge of which tissue belonged to which experimental group. Tissues taken for histopathological examination were fixed in 10 % of formaldehyde. Tissues obtained were washed under tap water for one night long and then were embedded into paraffin after being subject to routine histological procedures. Sections of 5 μm taken from the paraffin blocks were taken onto slides^24^. Tissue samples prepared were dyed with hematoxylin-eosin and evaluated under light microscope as shown in Table 1.

**Table 1:** Pathological evaluation criteria.

(0)	No injury healing/adherence
(+)	Anastomosis line is open from one or more points or low level of healing
(++)	No leakage in anastomosis, there is scar tissue and anastomosis ends are active (Edema in tissue, congestion, hypercellular scar reaction, mononuclear cellular infiltration)
(+++)	Anastomosis poles are active, there is granulation tissue in-between and healthier look
(++++)	Full injury healing, healthy look with epithelialisation

### *Statistical method*

Data obtained were indicated as average ± standard error (SE). Statistical significance levels of data were determined by using statistical packaged software “SPSS for Windows version 16” (Chicago, IL, USA). Multiple group comparisons were done by Kruskal-Wallis test. Mann Whitney U-test was used for comparison of two groups. For the interpretation of the result found, p<0.05 value was accepted as statistically significant.

## Results

No leakages were observed at the anastomosis area when abdomens of the rats were opened to take tissue samples after the anastomosis.

**Figure 1:**
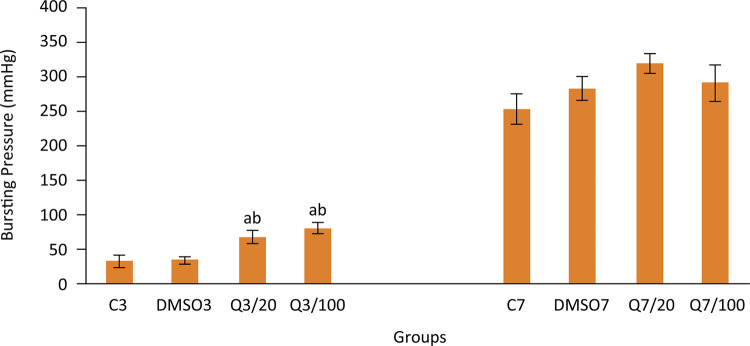
Bursting pressures in groups. (Group C3, control, anastomosis of 3 days; Group DMSO3, DMSO control, anastomosis of 3 days; Group Q3/20, quercetin 20 mg/kg, anastomosis of 3 days; Group Q3/100, quercetin 100 mg/kg, anastomosis of 3 days; Group C7, anastomosis of 7 days; Group DMSO7, DMSO control, anastomosis of 7 days; Group Q7/20, quercetin 20 mg/kg, anastomosis of 7 days; Group Q7/100, quercetin 100 mg/kg, anastomosis of 7 days. Columns show average and standard error. a: p<0.05 *vs.* group C3, b: p<0,05 *vs.* group DMSO3)

Bursting pressure levels (mmHg) in 3 days groups, which represent the early period of anastomosis injury healing, were recorded as 32.1±8.4, 33.5±5.1, 67.6±9.5, and 79.4±8.0 for groups C3, DMSO3, Q3/20, and Q3/100 respectively. Significantly higher bursting pressure levels in groups which were applied with quercetin were remarkable (p<0.05 quercetin treated groups versus control group). On the other hand, bursting pressure levels of all groups were similar 7 days after the operation (253.8±21.7, 284.27±17.2, 319.8±14.0, and 291.4±27.0 for groups C7, DMSO7, Q7/20, and Q7/100 respectively). Bursting pressure levels in early and late periods of injury healing are shown in Figure 1.

**Figure 2:**
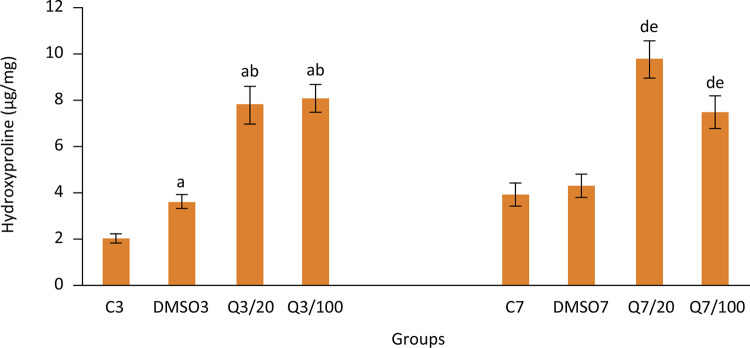
Hydroxyproline levels in groups. (Group C3, control, anastomosis of 3 days; Group DMSO3, DMSO control, anastomosis of 3 days; Group Q3/20, quercetin 20 mg/kg, anastomosis of 3 days; Group Q3/100, quercetin 100 mg/kg, anastomosis of 3 days; Group C7, anastomosis of 7 days; Group DMSO7, DMSO control, anastomosis of 7 days; Group Q7/20, quercetin 20 mg/kg, anastomosis of 7 days; Group Q7/100, quercetin 100 mg/kg, anastomosis of 7 days. Columns show average and standard error. a: p<0.05 *vs.* group C3, b: p<0.05 *vs.* group DMSO3, d: p<0.05 *vs.* group C7, e: p<0.05 *vs.* group DMSO7)

On the third day following the anastomosis, results obtained for hydroxyproline levels, which is an indicator of injury healing, were found to be similar with bursting pressure levels. Hydroxyproline levels (μg/mg ww) of scar tissue in groups which were applied with quercetin were significantly higher than groups which were not applied with quercetin (2.0±0.2, 3.6±0.3, 7.8±0.8, 8.1±0.6 for groups C3, DMSO3, Q3/20, Q3/100 respectively and 3.9±0.5, 4.3±0.5, 9.8±0.8, 7.5±0.7 for groups C7, DMSO7, Q7/20, Q7/100 respectively). Similarly, it can be seen in Figure 2 that quercetin significantly increased hydroxyproline level in the late period.

**Figure 3:**
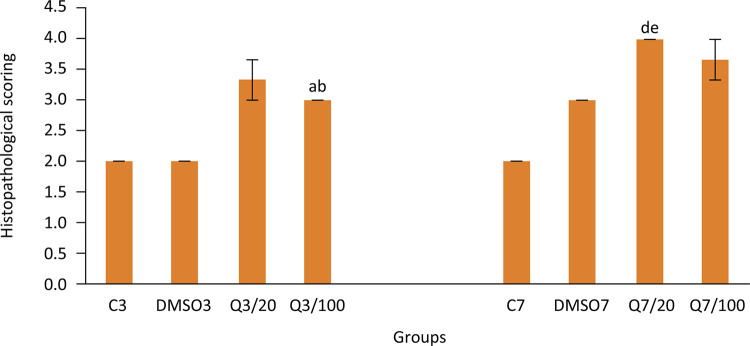
Histopathological scoring in groups. (Group C3, control, anastomosis of 3 days; Group DMSO3, DMSO control, anastomosis of 3 days; Group Q3/20, quercetin 20 mg/kg, anastomosis of 3 days; Group Q3/100, quercetin 100 mg/kg, anastomosis of 3 days; Group C7, anastomosis of 7 days; Group DMSO7, DMSO control, anastomosis of 7 days; Group Q7/20, quercetin 20 mg/kg, anastomosis of 7 days; Group Q7/100, quercetin 100 mg/kg, anastomosis of 7 days. Columns show average and standard error. a: p<0.05 *vs.* group C3, b: p<0.05 *vs.* DMSO3, d: p<0.05 *vs.* group C7, e: p<0.05 *vs.* group DMSO7)

Injury healing was also evaluated by using histopathological scoring. Histopathological healing score was found to be significantly higher in the group which was applied with 100 mg/kg quercetin on the third day following the anastomosis (Q3/100) compared to groups which were not applied with quercetin. On the other hand, histopathological healing score of group Q3/20, whose bursting pressure and hydroxyproline levels of scar tissue were significantly higher than the groups which were not applied with quercetin, was found to be higher than these groups but the difference was not statistically meaningful. Histopathological healing scores obtained on the seventh day of healing were significantly higher in Q7/20 group which were applied with quercetin compared to groups which were not applied with quercetin. However, a statistical significance could not be detected because of the high standard deviation in group Q7/100 (p= 0,068 versus group C7, p= 0,182 versus group DMSO7). Histopathological scores can be seen in Figure 3.

Injury healing is substantially affected by the equilibrium between the oxidant damage antioxidant defense systems. As a matter of fact, on the third day following the anastomosis, MDA levels of scar tissue, which is an indicator of oxidant damage, were found to be significantly higher in groups which were not applied with quercetin (22.2±2.2, 16.2±1.5, 5.8±0.5, 9.2±0.7 for groups C3, DMSO3, Q3/20, Q3/100 respectively and 13.6±1.3, 13.7±1.4, 6.2±0.6, 9.5±1.1 for groups C7, DMSO7, Q7/20, Q7/100 respectively). Application of quercetin decreased the oxidant damage by strengthening the antioxidant defense system in the injury site on the third day following the operation.

Catalase activity (U/mg ww) values obtained on the third day of the study were found to be 19.2±0.8, 22.6±1.1, 34.1±2.7, 35.9±4 for groups C3, DMSO3, Q3/20, Q3/100 respectively whereas SOD activity (U/mg ww) values were found to be 6.3±0.3, 7.7±0.4, 10.1±0.6, 10.7±0.4 for groups C3, DMSO3, Q3/20, Q3/100 respectively.

Furthermore, it is seen that the effect of quercetin on decreasing the oxidant damage during the late period of injury healing continues especially in group Q7/20. Catalase activity values were measured as 19.9±1.3, 26.3±1.5,39.6±3.5, 34.9±2.2 and SOD activity values were measured as 8.1±0.5, 8.6±0.4, 13.1±0.7, 9.4±0.4 for groups C7, DMSO7, Q7/20, Q7/100 respectively.

**Figure 4:**
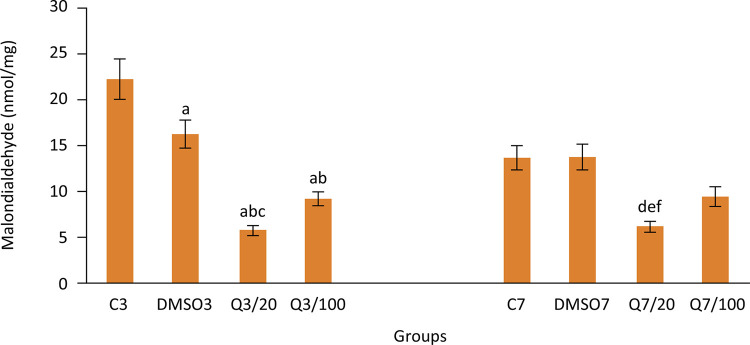
Malondialdehyde (MDA) levels in groups. (Group C3, control, anastomosis of 3 days; Group DMSO3, DMSO control, anastomosis of 3 days; Group Q3/20, quercetin 20 mg/kg, anastomosis of 3 days; Group Q3/100, quercetin 100 mg/kg, anastomosis of 3 days; Group C7, anastomosis of 7 days; Group DMSO7, DMSO control, anastomosis of 7 days; Group Q7/20, quercetin 20 mg/kg, anastomosis of 7 days; Group Q7/100, quercetin 100 mg/kg, anastomosis of 7 days. Columns show average and standard error. a: p<0.05 *vs.* group C3, b: p<0.05 *vs.* group DMSO3, c: p<0.05 *vs.* group Q3/100, d: p<0.05 *vs.* group C7, e: p<0.05 *vs.* group DMSO7, f: p<0.05 *vs.* group Q7/100)

**Figure 5 f05:**
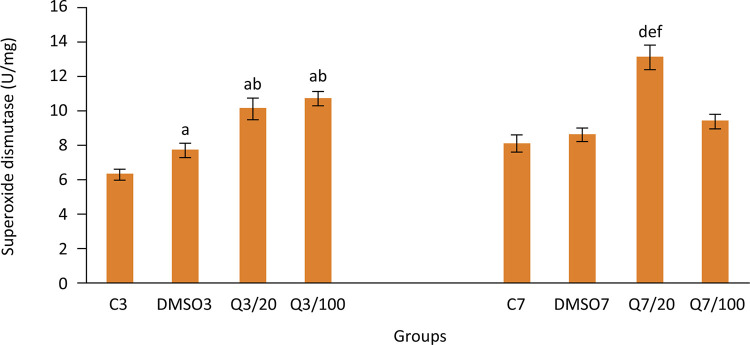
Superoxide dismutase (SOD) activities in groups. (Group C3, control, anastomosis of 3 days; Group DMSO3, DMSO control, anastomosis of 3 days; Group Q3/20, quercetin 20 mg/kg, anastomosis of 3 days; Group Q3/100, quercetin 100 mg/kg, anastomosis of 3 days; Group C7, anastomosis of 7 days; Group DMSO7, DMSO control, anastomosis of 7 days; Group Q7/20, quercetin 20 mg/kg, anastomosis of 7 days; Group Q7/100, quercetin 100 mg/kg, anastomosis of 7 days. Columns show average and standard error. a: p<0.05 *vs.* group C3, b: p<0.05 *vs.* group DMSO3, d: p<0.05 *vs.* group C7, e: p<0.05 *vs.* group DMSO7, f: p<0.05 *vs.* group Q7/100)

**Figure 6 f06:**
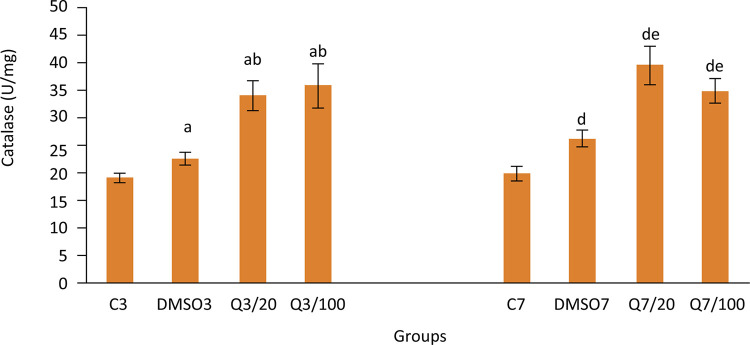
Catalase activities in groups. (Group C3, control, anastomosis of 3 days; Group DMSO3, DMSO control, anastomosis of 3 days; Group Q3/20, quercetin 20 mg/kg, anastomosis of 3 days; Group Q3/100, quercetin 100 mg/kg, anastomosis of 3 days; Group C7, anastomosis of 7 days; Group DMSO7, DMSO control, anastomosis of 7 days; Group Q7/20, quercetin 20 mg/kg, anastomosis of 7 days; Group Q7/100, quercetin 100 mg/kg, anastomosis of 7 days. Columns show average and standard error. a: p<0.05 *vs.* group C3, b: p<0.05 *vs.* group DMSO3, d: p<0.05 *vs.* group C7, e: p<0.05 *vs.* group DMSO7)

Data on oxidant damage and antioxidant defense systems of groups can be found in Figures 4 to 6 [Fig f05] [Fig f06].

## Discussion

These are the key results of our study: Quercetin is one of the factors that have a significantly curative effect on injury healing in the early period; it increases antioxidant capacity both in the early and late period and contributes to injury healing.

One of the measurement methods of injury strength in experimental anastomosis studies is bursting pressure. We also used bursting pressure for measuring injury strength in our study. In our study, high bursting pressures recorded 3 days after the anastomosis in groups to which quercetin was applied show that quercetin deliberately speeds the injury healing in this period. Histopathological data in Q3/20 and Q3/100 groups also support this finding. The fact that quercetin did not make a significant change in bursting pressure on the seventh day suggests that the effect of speeding the injury healing is more explicit in the early period.

One of the important factors in injury healing is reactive oxygen species (ROS). ROS is produced by nicotinamide adenine dinucleotide phosphate (NADPH) oxidase during normal metabolic incidents. Hydrogen peroxide (H_2_O_2_) is one of these and it is not a radical. However, it can cause serious damages in cells^25^. The existence of iron and copper ions in the environment causes H_2_O_2_ to form hydroxyl radicals. These radicals can cause cell damage. ROS is an important route to protect the injury area against pathogenic microorganisms. A major part of ROS formation is realized by neutrophils and macrophages. ROS can cause damages in the surrounding tissues along with removal of microorganisms in the environment^26^. Superoxide anion (O_2_^-^) originated subject to inflammation of endothelial cells in the injury area and H_2_O_2_ stimulate neovascularization by regulating micro circulation. By this means, nutrition and oxygen need of the injury area are met. It was also stated that ROS in low levels has a role in signal transmission in cells^27^. Oxidants and free radicals in the injury area can deteriorate healing by causing tissue damage. Major factors of this deterioration are O_2_^-^anion and hydroxyl radicals. They crash the proline and hydroxyproline which constitute the collagen and can change the adhesion, reproduction, and vitality of fibroblasts. Nonetheless, they cause serious damages in fibroblasts by inhibiting the migration of H_2_O_2_ keratinocytes and signal transmission of epidermal growing factor (EGF)^28^. High level of ROS and increased inflammatory infiltration in the injury area indicate the existence of oxidative stress. Increase in ROS level causes cytotoxicity and delays injury healing. For this reason, reducing the effects of ROS is very critical for injury healing. Thus, the antioxidant substances stand out. Today antioxidants are used for injury healing^29^. There are several studies that examine the healing of injuries by using antioxidants^19,20,30^.

In Kabali *et al*.^31^, which is one of these studies, injury healing by applying acetylcysteine to the injury area in colon anastomosis in rats was examined. A significant acceleration in injury healing in rats that were treated with acetylcysteine was seen. Çakmak *et al*.^19^ found out in their studies that resveratrol and simvastatin speed up the healing process of colon anastomosis injuries. In these studies, it is observed that acceleration of injury healing is accompanied by the decrease in oxidant damage.

Gopalakrishnan *et al*.^9^ examined the effects of quercetin on injury healing in a study. It was seen that quercetin significantly increased vascular endothelial growth factor (VEGF) and transforming growth factor beta-1 (TGFβ-1) expressions, which are important in injury healing, and decreased tumor necrosis factor alpha (TNF-α) expression. Nonetheless, it was also stated that it decreased the number of inflammatory cells, increased fibroblast proliferation and microvessel density, and regulated reepithelization and collagen accumulation. In our study, it was seen that quercetin caused a significant change in hydroxyproline levels at a dose of 20 mg/kg and 100 mg/kg in short and long period and contributed to injury healing.

One of the most important components of the colorectal wall is a lymphoid structure which is called the Peyer’s plaque. The Peyer’s plaque can be found in different locations in male and female genders. The collagen content of the Peyer’s plaque is lower compared to other parts of the colon. The collagen amount can affect the healing process of the anastomosis line^32^. We did not take into consideration the location of the Peyer’s plaque while performing the resection and end-to-end anastomosis. However, the probable disadvantages which could be caused by the location of the Peyer’s plaque were minimized by the facts that we used rats of only one type of gender (male) and also we performed the surgical protocol on the exact same anatomic area in all animals.

Gomathi *et al*.^7^ examined the effects of a biomaterial, which is a compound of quercetin and collagen matrix, on skin injuries. It was reported that quercetin increases cell proliferation, hydroxyproline levels, injury healing and decreases levels of free radicals by showing antioxidant effect. According to these results, they suggested that quercetin could be used as a material for injury healing.

In our study, we observed that quercetin application increased SOD and catalase activities. Quercetin application for 3 days significantly increased the SOD activities in intestinal tissue. We reached similar results for CAT activities as well. By looking at these results, it can be said that quercetin application for 3 days, even with low doses, increases antioxidant capacity. In compliance with these results, MDA levels -an indicator of oxidant damage- were determined to be lower in Q3/20 and Q3/100 groups. Quercetin application for 3 days speeded the injury healing and meanwhile limited the oxidant damage by increasing the antioxidant capacity. By taking into consideration the studies which show that oxidant damage negatively affects injury healing, it is possible to tell that quercetin increases the injury site strength after anastomosis by improving the antioxidant capacity.

One of the interesting results of our study is that MDA levels of groups which were administered with quercetin at a dose of 20 mg/kg were lower than groups which were administered with quercetin at a dose of 100 mg/kg. This finding is coherent with lower SOD level determined in group Q7/20 compared to group Q7/100. It might be expected that a higher dose of quercetin would affect more both the oxidant damage and the antioxidant capacity. However, in the study of Kay *et al*.^33^ it was clearly shown that dose-response curves of flavonoids could be non-linear. Therefore, for using flavonoids as complementary treatment, more detailed studies on dose-response curves are needed.

Although antioxidant capacity in groups which were applied with quercetin for 7 days increased as well, it can be thought that quercetin is less effective in the late period compared to the early period by taking into consideration other factors involved that affect injury healing. Taking into account the major problems that anastomosis leakages cause especially in early periods, healing effect of quercetin in the early period is precious.

In a study about ischemic reperfusion damage, Ekingen *et al*.^34^ examined how the biochemical changes that occur in the early and late periods of reperfusion affected anastomosis healing. They found that the SOD enzyme activity, which is measured for evaluating the effect of ischemic reperfusion on the intestinal wall, increased significantly in the early period of the reperfusion compared to the control group. However, they observed that on the 24^th^ hour of the reperfusion, SOD enzyme activity decreased to lower levels compared to the control group. Lower SOD levels than the control group in the late period and non-existence of histopathological damage suggest that antioxidant effect was completed in the early period.

As a matter of fact, we used DMSO as a solvent in this study. Nonetheless, we obtained interesting results regarding particularly oxidant damage and antioxidant capacity in groups, which were administered with DMSO only (groups DMSO3 and DMSO7). The MDA level, which is accepted as an indicator of oxidative damage, of the groups which were administered with DMSO only (groups DMSO3 and DMSO7) was lower than the MDA level of the C3 group which took no treatment. Furthermore, both SOD and CAT activities of the DMSO3 group were found to be higher than the C3 group. We did not obtain such striking results in DMSO7 group in terms of oxidant damage and antioxidant capacity. We only detected an increase in CAT activity in this group. Similar to our study, there are several studies in the literature in which the antioxidant characteristic of the DMSO is emphasized^35-37^. In the study of Li *et al*.^35^, which is an example of these studies, DMSO showed a protective effect in gut barrier by increasing the SOD activity and decreasing the MDA level in gut barrier dysfunction generated by zymosan. At the same time, there are other studies in the literature in which the antioxidant effects of DMSO could not be determined^38,39^. Different studies are needed to understand the effects of DMSO on oxidative system. By considering the results of our study, it is possible to say that DMSO can augment the antioxidant effects of quercetin.

In our study, finding higher bursting pressure levels in Q3/20 and Q3/100 groups, which were formed to determine the effect of quercetin on injury healing in the early period, compared to C3 and DMS03 groups is an indicator of fine injury strength. Higher hydroxyproline levels in Q3/20 and Q3/100 groups compared to C3 and DMS03 groups coincide with and support the bursting pressure values. Quercetin did not have a significant effect on bursting pressures in the long term but hydroxyproline levels increased significantly in Q7/20 and Q7/100 groups compared to C7 group. Along with hydroxyproline, some other factors also play a role in injury site healing. Madden and Smith stated that knowing only the collagen amount was not enough to express the importance of collagen in injury site healing, it was also needed to know the collagen construction and destruction ratios^40^.

## Conclusions

The intraperitoneal application of quercetin dissolved in DMSO improves healing and reduces levels of tissue oxidative stress in colorectal anastomoses performed in rats. It is seen that quercetin contributes to healing of anastomosis injuries in the early period. It also regulates the oxidant and antioxidant parameters both in the early and the late period. According to these results, it can be suggested that quercetin accelerates the injury healing by improving antioxidant capacity.

In accordance with the results of our study, it is possible to say that quercetin can be useful in the healing of anastomosis injuries. It can be said that the antioxidant effect of quercetin is crucial in revealing this healing effect. However, additional studies are needed to know how quercetin affects molecular mechanisms in the healing process and by which paths it realizes this.

## References

[1] Bray F, Ferlay J, Soerjomataram I, Siegel RL, Torre LA, Jemal A (2018). Global cancer statistics 2018: GLOBOCAN estimates of incidence and mortality worldwide for 36 cancers in 185 countries. CA Cancer J Clin.

[2] Kirchhoff P, Clavien PA, Hahnloser D (2010). Complications in colorectal surgery: risk factors and preventive strategies. Patient Saf Surg.

[3] Vasiliu EC, Zarnescu NO, Costea R, Neagu S (2015). Review of risk factors for anastomotic leakage in colorectal surgery. Chirurgia.

[4] Gessler B, Eriksson O, Angenete E (2017). Diagnosis, treatment, and consequences of anastomotic leakage in colorectal surgery. Int J Colorectal Dis.

[5] Bielecki K, Gajda A (1999). The causes and prevention of anastomotic leak after colorectal surgery. Klinicka Onkologie Zvlastni Cislo.

[6] Soetersa PB, Zoeteta J, Dejonga CH, Williams NS, Baetena C (2002). Colorectal surgery and anastomotic leakage. Dig Surg.

[7] Gomathi K, Gopinath D, Rafiuddin Ahmed M, Jayakumar R (2003). Quercetin incorporated collagen matrices for dermal wound healing processes in rat. Biomaterials.

[8] Ahmad M, Sultana M, Raina R, Pankaj NK, Verma PK, Prawez S (2017). Hypoglycemic, hypolipidemic, and wound healing potential of quercetin in streptozotocin-induced diabetic rats. Pharmacogn Mag.

[9] Gopalakrishnan A, Ram M, Kumawat S, Tandan S, Kumar D (2016). Quercetin accelerated cutaneous wound healing in rats by increasing levels of VEGF and TGF-β1. Indian J Exp Biol.

[10] Formica JV, Regelson W (1995). Review of the biology of quercetin and related bioflavonoids. Food Chem Toxicol.

[11] Morel I, Lescoat G, Cogrel P, Sergent O, Pasdeloup N, Brissot P, Cillard P, Cillard J (1993). Antioxidant and iron-chelating activities of the flavonoids catechin, quercetin, and diosmetin on iron-loaded rat hepatocyte cultures. Biochem Pharmacol.

[12] Xu D, Hu MJ, Wang YQ, Cui YL (2019). Antioxidant activities of quercetin and its complexes for medicinal application. Molecules.

[13] Owumi SE, Danso OF, Effiong ME (2019). Dietary quercetin abrogates hepatorenal oxidative damage associated with dichloromethane exposure in rats. Acta Biochim Pol.

[14] Lyra HF, Lucca Schiavon L, Rodrigues IK, Couto Vieira DS, Paula Martins R, Turnes BL, Latini AS, D’Acâmpora AJ (2019). Effects of ghrelin on the oxidative stress and healing of the colonic anastomosis in rats. J Surg Res.

[15] Hajji S, Khedir SB, Hamza-Mnif I, Hamdi M, Jedidi I, Kallel R, Boufi S, Nasri M (2019). Biomedical potential of chitosan-silver nanoparticles with special reference to antioxidant, antibacterial, hemolytic and in vivo cutaneous wound healing effects. Biochim Biophys Acta Gen Subj.

[16] Choi BS, Song HS, Kim HR, Park TW, Kim TD, Cho BJ, Kim CJ, Sim SS (2009). Effect of coenzyme Q10 on cutaneous healing in skin-incised mice. Arch Pharm Res.

[17] Barlas AM, Kuru S, Kismet K, Cavusoglu T, Bag YM, Senes M, Cihan N, Celepli P, Unal Y, Hucumenoglu S (2018). Rectal application of argan oil improves healing of colorectal anastomosis in rats. Acta Cir Bras.

[18] Ersoy ÖF, Özkan N, Özsoy Z, Kayaoğlu HA, Yenidoğan E, Çelik A, Özuğurlu AF, Arabacı Çakır E, Lortlar N (2016). Effects of melatonin on cytokine release and healing of colonic anastomoses in an experimental sepsis model. Ulus Travma Acil Cerrahi Derg.

[19] Cakmak GK, Irkorucu O, Ucan BH, Tascilar O, Emre AU, Karakaya K, Bahadir B, Acikgoz S, Pasaoglu H, Ankarali H, Ugurbas E, Demirtas C, Comert M (2009). The effects of resveratrol on the healing of left colonic anastomosis. J Invest Surg.

[20] Cakmak GK, Irkorucu O, Ucan BH, Emre AU, Bahadır B, Demirtas C, Tascilar O, Karakaya K, Acikgoz S, Kertis G, Ankarali H, Pasaoglu H, Comert M (2009). Simvastatin improves wound strength after intestinal anastomosis in the rat. J Gastrointest Surg.

[21] Nassiri-Asl M, Moghbelinejad S, Abbasi E, Yonesi F, Haghighi MR, Lotfizadeh M, Bazahang P (2013). Effects of quercetin on oxidative stress and memory retrieval in kindled rats. Epilepsy Behav.

[22] Zhang W, Wang Y, Yang Z, Qiu J, Ma J, Zhao Z, Bao T (2011). Antioxidant treatment with quercetin ameliorates erectile dysfunction in streptozotocin-induced diabetic rats. J Biosci Bioeng.

[23] Ohkawa H, Ohishi N, Yagi K (1979). Assay of lipid peroxides in animal tissues by thiobarbituric acid reaction. Anal Biochem.

[24] Chiu CJ, McArdle AH, Brown R, Scott HJ, Gurd FN (1970). Intestinal mucosal lesions in low-flow states. I. A morphological, hemodynamic, and metabolic reappraisal. Arch Surg.

[25] Hensley K, Robinson KA, Gabbita SP, Salsman S, Floyd RA (2000). Reactive oxygen species, cell signaling, and cell injury. Free Radic Biol Med.

[26] Bayır H (2005). Reactive oxygen species. Crit Care Med.

[27] Blokhina O, Virolainen E, Fagerstedt KV (2003). Antioxidants, oxidative damage and oxygen deprivation stress: a review. Ann Bot.

[28] Yager DR, Kulina RA, Gilman LA (2007). Wound fluids: a window into the wound environment?. Int J Low Extrem Wounds.

[29] Moradi M, Moradi A, Alemi M, Ahmadnia H, Abdi H, Ahmadi A, Bazargan-Hejazi S (2010). Safety and efficacy of clomiphene citrate and L-carnitine in idiopathic male infertility: a comparative study. Urol J.

[30] Zeytin K, Çiloğlu NS, Ateş F, Aker VF, Ercan F (2014). Resveratrolün diyabetik sıçanlarda tendon iyileşmesi üzerine etkileri. Acta Orthop Traumatol Turc.

[31] Kabali B, Girgin S, Gedik E, Ozturk H, Kale E, Buyukbayram H (2009). N-Acetylcysteine prevents deleterious effects of ischemia/reperfusion ınjury on healing of colonic anastomosis in rats. Eur Surg Res.

[32] Priolli DG, Eiras da Silva PL, Betini AM, Pereira JA, Margarido NF, Real Martinez CA (2009). Is peritoneal reflection the best anatomical repair landmark in experimental colorectal surgery on rats?. Acta Cir Bras.

[33] Kay CD, Hooper L, Kroon PA, Rimm EB, Cassidy A (2012). Relative impact of flavonoid composition, dose and structure on vascular function: a systematic review of randomised controlled trials of flavonoid-rich food products. Mol Nutr Food Res.

[34] Ekingen G, Ceran C, Demirtola A, Demiroğulları B, Sancak B, Poyraz A, Sonmez K, Basaklar AC, Kale N (2006). İnce Barsak İskemi Reperfüzyonunda Reperfüzyon Süresinin Biyokimyasal Değişiklikler ve Anastomoz İyileşmesine Etkisi. İnönü Üniversitesi Tıp Fakültesi Dergisi.

[35] Li YM, Wang HB, Zheng JG, Bai XD, Zhao ZK, Li JY, Hu S (2015). Dimethyl sulfoxide inhibits zymosan-induced intestinal inflammation and barrier dysfunction. World J Gastroenterol.

[36] Boybeyi O, Bakar B, Aslan MK, Atasoy P, Kisa U, Soyer T (2014). Evaluation of dimethyl sulfoxide and dexamethasone on pulmonary contusion in experimental blunt thoracic trauma. Thorac Cardiovasc Surg.

[37] Ueda T, Toyoshima Y, Kushihashi T, Hishida T, Yasuhara H (1993). Effect of dimethyl sulfoxide pretreatment on activities of lipid peroxide formation, superoxide dismutase and glutathione peroxidase in the mouse liver after whole-body irradiation. J Toxicol Sci.

[38] Akang EN, Dosumu OO, Afolayan OO, Fagoroye AM, Osiagwu DD, Usman IT, Oremosu AA, Akanmu AS (2019). Combination antiretroviral therapy (cART)-induced hippocampal disorders: highlights on therapeutic potential of Naringenin and Quercetin. IBRO Rep.

[39] Ali BH, Mousa HM (2001). Effect of dimethyl sulfoxide on gentamicin-induced nephrotoxicity in rats. Hum Exp Toxicol.

[40] Madden JW, Smith HC (1970). The rate of collagen synthesis and deposition in dehisced and resutured wounds. Surg Gynecol Obstet.

